# Neutrophils and COVID-19: Active Participants and Rational Therapeutic Targets

**DOI:** 10.3389/fimmu.2021.680134

**Published:** 2021-06-02

**Authors:** Jon Hazeldine, Janet M. Lord

**Affiliations:** ^1^ MRC-Versus Arthritis Centre for Musculoskeletal Ageing Research, Institute of Inflammation and Ageing, University of Birmingham, Birmingham, United Kingdom; ^2^ National Institute for Health Research Surgical Reconstruction and Microbiology Research Centre, Queen Elizabeth Hospital Birmingham, Birmingham, United Kingdom; ^3^ National Institute for Health Research Birmingham Biomedical Research Centre, University Hospital Birmingham NHS Foundation Trust and University of Birmingham, Birmingham, United Kingdom

**Keywords:** COVID-19, immune dysregulation, neutrophils, SARS-CoV-2, neutrophil extracellular traps

## Abstract

Whilst the majority of individuals infected with severe acute respiratory syndrome coronavirus 2 (SARS-CoV-2), the causative pathogen of COVID-19, experience mild to moderate symptoms, approximately 20% develop severe respiratory complications that may progress to acute respiratory distress syndrome, pulmonary failure and death. To date, single cell and high-throughput systems based analyses of the peripheral and pulmonary immune responses to SARS-CoV-2 suggest that a hyperactive and dysregulated immune response underpins the development of severe disease, with a prominent role assigned to neutrophils. Characterised in part by robust generation of neutrophil extracellular traps (NETs), the presence of immature, immunosuppressive and activated neutrophil subsets in the circulation, and neutrophilic infiltrates in the lung, a granulocytic signature is emerging as a defining feature of severe COVID-19. Furthermore, an assessment of the number, maturity status and/or function of circulating neutrophils at the time of hospital admission has shown promise as a prognostic tool for the early identification of patients at risk of clinical deterioration. Here, by summarising the results of studies that have examined the peripheral and pulmonary immune response to SARS-CoV-2, we provide a comprehensive overview of the changes that occur in the composition, phenotype and function of the neutrophil pool in COVID-19 patients of differing disease severities and discuss potential mediators of SARS-CoV-2-induced neutrophil dysfunction. With few specific treatments currently approved for COVID-19, we conclude the review by discussing whether neutrophils represent a potential therapeutic target for the treatment of patients with severe COVID-19.

## Introduction

Severe acute respiratory syndrome coronavirus 2 (SARS-CoV-2), an enveloped, single-stranded positive-sense RNA virus, is the causative pathogen of Coronavirus disease 2019 (COVID-19), a respiratory tract infection that was declared a global pandemic by The World Health Organisation on March 11^th^ 2020 (1). Respiratory droplets and direct contact with infected individuals are the main routes of transmission for SARS-CoV-2, which was first identified in bronchoalveolar-lavage fluid (BALF) samples obtained from patients admitted to the Wuhan Jinyuntan Hospital in the Hubei Province of China with severe pneumonia of unknown aetiology in December 2019 (2). As of 10^th^ May 2021, the virus has infected over 157 million people worldwide, resulting in over 3.2 million deaths (3). Whilst the majority of individuals (~80%) infected with SARS-CoV-2 experience a self-resolving mild to moderate respiratory disease, 10-20% of patients diagnosed with COVID-19 develop a severe viral pneumonia (4). In approximately 5% of cases, disease progresses to a critical illness characterised by acute respiratory distress syndrome (ARDS) and respiratory failure that requires ventilatory support and specialised treatment in intensive care units (ICU) (4–8). Through systematic reviews and meta-analyses, a number of demographic and host risk factors have been identified that influence the clinical outcomes of COVID-19 patients. Linked to reduced physiological reserve, a heightened basal inflammatory state or systemic endothelial dysfunction, advanced age, male gender and pre-existing co-morbidities [e.g. diabetes, malignancy, hypertension, cardiovascular disease and chronic kidney disease (CKD)] have been shown to be associated with increased disease severity, admission to ICU and mortality (9–12). However, as adults aged 20-44 years account for 20% and 12% of COVID-19-associated hospitalisations and ICU admissions respectively (13), and >5% of hospitalised younger adults (aged 18-34 years) with no underlying health conditions experience respiratory failure (14), additional host and/or virus-associated factors must contribute to disease pathogenesis. A dysregulated physiological response to SARS-CoV-2 infection is one such factor, with striking differences observed in the immune response of COVID-19 patients when graded by disease severity, with a profound inflammatory and dysregulated myeloid response emerging as a key driver of severe COVID-19 (15–21).

Integral to the initiation of anti-viral immune responses is the production of type I (α/β) and type III (γ) interferons (IFNs) (22). Compared to those with mild/moderate disease, patients with critical COVID-19 exhibit an impaired peripheral type I IFN response (23). Shown to precede the onset of respiratory failure, this weakened IFN response results in an increased plasma viral load (23) that triggers excessive nuclear factor kappa β-driven inflammatory responses, a hallmark of severe COVID-19 (23–25). Indeed, significantly elevated concentrations of interleukin (IL)-2, IL-6, IL-7, IL-8, IL-10, IL-18, tumour necrosis factor-alpha (TNF-α), granulocyte colony stimulating factor (G-CSF), monocyte chemoattractant protein 1 (MCP-1) and macrophage inflammatory protein 1 alpha (MIP-1α) have been measured in the circulation of ICU patients when compared to their non-ICU counterparts (5, 26–29), with additional studies reporting significantly higher concentrations of IL-6 in non-survivors versus survivors (30, 31). Reminiscent of a cytokine storm, this hyperinflammatory state would be exacerbated by the systemic dysregulation in lipid metabolism that occurs in patients with severe COVID-19 (32). Compared to non-ICU patients, significantly reduced circulating concentrations of the proresolving lipid mediator resolvin E3 have been detected in ICU patients, who also present with increased concentrations of pro-inflammatory lipids generated by the enzymes ALOX5 and cytochrome p450 (32). Linked to this state of systemic hyperinflammation are aberrant SARS-CoV-2-induced innate and adaptive immune responses. Lymphopenia, T cell exhaustion, impaired activity of cytotoxic lymphocytes, emergency granulopoiesis and increased neutrophil activation are just some of the examples of immune dysregulation that have been reported in patients with severe COVID-19 (28, 33–35). Of these, it is the systemic hyperactivation of neutrophils, and their infiltration into pulmonary tissues, that has been assigned a prominent role in the immunopathology that underpins the transition from mild to severe COVID-19 (35–37).

**Figure 1:**
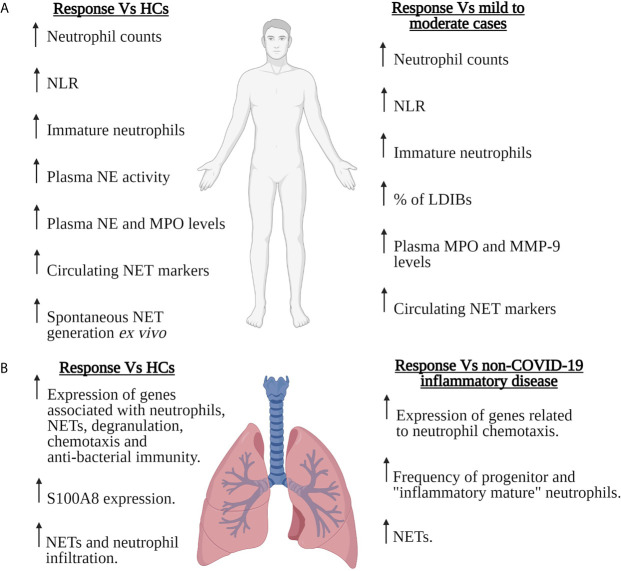
Neutrophils in severe COVID-19. Summary of the SARS-CoV-2-induced changes that have been reported for the number, composition and/or function of circulating **(A)** or pulmonary **(B)** neutrophils in patients with severe COVID-19. Features of the neutrophil response of severe COVID-19 patients are compared to those of healthy controls (HCs), patients with mild-to-moderate COVID-19 or patients with non-COVID-19 inflammatory disease. For the pulmonary neutrophil response summarised in **(B)**, data was derived from the analysis of bronchoalveolar lavage fluid or post-mortem lung tissue sections. LDIBs, Low density inflammatory band cells; MMP-9, Matrix metalloproteinase-9; MPO, Myeloperoxidase; NE, Neutrophil elastase; NETs, Neutrophil extracellular traps; NLR, Neutrophil to lymphocyte ratio. Figure created with BioRender.com.

Renowned for providing immediate frontline protection against rapidly dividing bacteria, fungi and yeast, there is a growing body of evidence implicating neutrophils, via their generation of reactive oxygen species (ROS), neutrophil extracellular traps (NETs) and ability to act as antigen presenting cells, in the host response to viral infections (38, 39). However, their involvement must be tightly regulated as exaggerated neutrophil responses to viral challenge result in tissue injury, vascular leakage and organ dysfunction (38, 39). In the context of SARS-CoV-2, cross-sectional and prospective studies analysing peripheral blood, BALF and/or lung tissue samples from COVID-19 patients of differing disease severities have demonstrated significant virus-induced changes in the composition, phenotype and/or function of circulating and pulmonary neutrophil pools (**Figure 1**). Neutrophilia, robust NET generation and the presence of immature, immunosuppressive and pro-inflammatory neutrophil subsets are examples of some of the consequences of SARS-CoV-2 infection described thus far, with data suggesting that signatures of neutrophil activation are enriched in patients with severe COVID-19 and associated with poor clinical outcome (17–21). Combined with the results of rodent and non-human primate based studies, these data demonstrate striking similarities between SARS-CoV-2-induced changes in neutrophil biology and the neutrophil response triggered by other members of the Coronaviridae family such as Middle East respiratory syndrome coronavirus (MERS-CoV) and SARS-CoV (**Table 1**).

**Table 1:** Changes reported in the number/frequency of circulating or pulmonary neutrophils in patients, rodents or non-human primates infected with SARS-CoV, SARS-CoV-2 or MERS and their relationship with disease severity or clinical outcome.

	SARS-CoV	SARS-CoV-2	MERS-CoV
**Blood**	**Humans** Increased neutrophil/granulocyte counts and/or frequencies when compared to reference ranges (40, 41). Increased neutrophil count at hospital presentation is associated with ICU admission or death (42). Neutrophilia during hospitalisation is associated with an increased incidence of hospital-acquired bacterial infections (41). High neutrophil count at hospital admission is an independent predictor of severe illness (43). Increased neutrophil count in intubated Vs non-intubated patients (44). **Rodents** Increased neutrophil frequency when compared to pre-infection levels (45).	**Humans** Increased neutrophil counts and/or frequencies when compared to HCs (28, 29, 46, 47). Increased neutrophil counts in severe COVID-19 patients when compared to those with mild to moderate disease (48–51). COVID-19 non-survivors present at hospital admission with significantly elevated neutrophil numbers (52–54). High neutrophil count at hospital admission is an independent predictive factor for in-hospital death (55). Elevated neutrophil counts at hospital admission reported for patients who are later admitted to ICU (6). Neutrophilia is a risk factor for the development of ARDS and for the progression of ARDS to death (56).	**Humans** Increased neutrophil counts when compared to reference ranges (57, 58). Increased neutrophil counts in severe Vs. mild cases (59). Increased neutrophil count in non-survivors Vs. survivors (60). **Rodents** Increased neutrophil frequency and counts when compared to non-infected littermates (61). Neutrophil counts increase with disease severity (61). **Non-human primates** Increased neutrophil number post-infection (62).
**Lungs**	**Humans** Neutrophilic infiltrates in the lung (63). **Rodents** Increased neutrophil frequency in the lungs of infected mice (64).	**Humans** Intense neutrophilic infiltrate within the alveolar spaces and/or pulmonary vessels (37, 46, 65–68). **Non-human primates** Neutrophils accumulate in bronchial lumens, alveolar spaces and pulmonary lesions (69–72).	**Humans** High numbers of neutrophils detected in BALF samples (73). **Non-human primates** Neutrophil infiltration into the lungs (62, 74, 75).

ARDS, Acute respiratory distress syndrome; BALF, Bronchoalveolar lavage fluid; CoV, Coronavirus; COVID-19, Coronavirus disease 2019; HCs, Healthy controls; ICU, Intensive care unit; MERS-CoV, Middle East respiratory syndrome coronavirus; SARS, Severe acute respiratory syndrome.

Here, we provide a comprehensive overview of the changes that have been described in the composition, phenotype and function of the neutrophil pool in COVID-19 patients of differing disease severities, and discuss potential mediators of SARS-CoV-2-induced neutrophil dysfunction. With no specific anti-viral therapies currently approved for the treatment of SARS-CoV-2 infection, we conclude the review by discussing whether suppressing the production of or enhancing the clearance of NETs, or suppressing the release of immature neutrophils from the bone marrow represent viable therapeutic strategies for the treatment of severe COVID-19.

## Neutrophils and COVID-19 Pathobiology – Animal Model Data

Animal models of infection provide valuable insights into the cellular and molecular mechanisms that underpin disease pathogenesis. Reminiscent of the pulmonary pathologies described for human COVID-19 patients with mild to severe disease, SARS-CoV-2 infection of non-human primates (e.g. African green monkeys, rhesus macaques, baboons and marmosets) results in lung consolidation, pulmonary edema, bronchointerstitial pneumonia, pleural adhesions, interstitial inflammation and diffuse alveolar damage (69–71, 76, 77). Across this spectrum of disease, analysis of post-mortem lung tissue has revealed that neutrophils accumulate in bronchial lumens, alveolar spaces and pulmonary lesions (69–72), with this infiltration coinciding with significantly elevated BALF concentrations of the chemokine IL-8 (70). Moreover, mirroring the changes reported in the composition of the pulmonary neutrophil pool in humans with severe COVID-19 (48, 78) immature “pre-neutrophils” have been detected in the lung sections of African green monkeys with high SARS-CoV-2 viral loads (79).

Angiotensin-converting enzyme 2 (ACE2), a type I integral membrane glycoprotein, is the receptor utilised by SARS-CoV-2 for cell entry (80). Due to the presence of ACE2 on the surface of cells that line their respiratory tract, ferrets have also emerged as a suitable host in which to study the pathogenesis of COVID-19 (81–83). Histological examination of lung tissue from SARS-CoV-2 infected ferrets has detected the presence of neutrophils in the alveolar septa, alveolar lumen and bronchiolar luminae (81, 83). Associated with bronchopneumonia, this neutrophilic infiltrate was observed primarily in ferrets infected with high doses of SARS-CoV-2 (81).

By replicating many of the pathological features observed in human disease, animal models of SARS-CoV-2 infection have been used to test potential therapies for the treatment of COVID-19 (71, 84, 85). Using a rhesus macaque infection model, Hoang et al showed that daily treatment with baricitinib, a selective JAK kinase inhibitor, significantly reduced the severity of SARS-CoV-2 infection (84). Associated with a milder inflammatory response, the beneficial effects of baricitinib treatment were attributed in part to reduced neutrophil-mediated immunopathology (84). For example, bulk RNA sequencing (RNA-seq) of BAL revealed a baricitinib-induced downregulation of genes associated with neutrophil degranulation, chemotaxis and extravasation (84). In line with these observations, flow cytometry analysis of BAL revealed significantly lower frequencies of neutrophils in baricitinib treated macaques when compared to untreated controls (84). Furthermore, baricitinib treatment was associated with reduced *ex vivo* NET formation and a virtual absence of NETs in post-mortem lung tissue (84). Pointing towards a prominent role for neutrophils in the pathology of severe COVID-19, these baricitinib-induced changes in neutrophil number and function coincided with a reduction in pulmonary lesions and lung pathology scores (84). Taken together, these findings offer potential mechanistic explanations for the improved clinical outcomes that have been reported for severe COVID-19 patients treated with baricitinib (86, 87).

## The Neutrophil Response to SARS-CoV-2 Infection

### Neutrophils in Blood

#### Neutrophil Number and the Neutrophil:Lymphocyte Ratio

The majority of studies that have compared full blood counts between COVID-19 patients and healthy controls (HCs) have reported a SARS-CoV-2-induced increase in the absolute number and/or frequency of circulating neutrophils (28, 29, 46–49). However, when accounting for disease severity, neutrophilia is not observed amongst all patient groups. For example, across several studies, no significant differences were reported in either the number or percentage of neutrophils between COVID-19 patients with mild or moderate disease and HCs (48–50). In contrast, severe COVID-19 patients exhibit neutrophil counts that are significantly greater than those recorded for both HCs and non-ICU patients with mild to moderate disease (48–51, 88–96). The exaggerated hyperinflammatory response that occurs in severe COVID-19 patients (5), particularly the systemic elevation in IL-6, has been suggested to drive this relationship between disease severity and elevated neutrophil counts (6, 15).

A handful of longitudinal studies that have performed serial sampling on hospitalised patients (51, 95, 97, 98) have shown the severity associated increase in neutrophil number persists over time. For example, across seven study time-points, ranging from days 1-15 post-hospitalisation, Ding et al observed significantly higher neutrophil counts in severe COVID-19 patients when compared to those with non-severe disease (51). Moreover, paired sample analyses have shown no significant difference in neutrophil counts between the hospital admission blood samples of severe COVID-19 patients, where neutrophilia is observed, and those obtained prior to discharge, suggesting a state of unresolved inflammation at the time of clinical recovery (95).

As patients with severe COVID-19 present at hospital admission with significantly higher neutrophil counts than those with mild to moderate disease (49, 51, 89, 91–93, 99–101), a number of studies have investigated whether neutrophilia at presentation can be used to assist in the diagnosis of severe COVID-19. Using a range of optimal cut off values (3.2-7.305 x 10^9^/L), it has been demonstrated that neutrophil counts can discriminate non-severe from severe COVID-19 patients with a degree of accuracy ranging from poor to excellent (88, 95, 96, 102). In a meta-analysis of 26 studies, Soraya et al reported that a cut-off value of 3.65 x 10^9^/L provided fair discrimination between severe and non-severe cases (AUROC, 0.79) (99). However, as observed in other meta-analyses (103), this model was outperformed by those built on lymphocyte counts or concentrations of acute phase proteins (99), suggesting that other clinical indices may be better placed to aid clinicians in the early identification of severe COVID-19 patients.

Relative to HCs, hospitalised COVID-19 patients, of all disease severities, exhibit significantly reduced numbers of circulating lymphocytes (28, 47, 49, 89). However, as with neutrophilia, the degree of lymphopenia is markedly influenced by disease severity, with severe COVID-19 patients presenting with significantly lower lymphocyte counts than those with mild to moderate disease (49–51, 89, 90, 92, 94–96, 100, 104). Suggested to be a consequence of systemic inflammation and/or SARS-CoV-2-induced cell death, this state of lymphopenia, when combined with maintained or elevated neutrophil counts, results in an elevated neutrophil:lymphocyte ratio (NLR) (89, 105). An increased NLR at hospital admission is emerging as a useful indicator of disease severity and a potential tool of diagnostic and prognostic utility (106, 107). For example, significantly higher baseline NLR values have been measured in COVID-19 patients who develop severe/critical disease when compared to mild or moderate cases (51, 90, 92, 95, 108–111), with this elevation persisting for up to 15 days post-hospital admission (51, 100, 112). In terms of its discriminatory power, NLR has been shown to distinguish severe COVID-19 patients from non-severe cases with a level of accuracy considered fair to good based on AUROC values (95, 100, 107, 109, 112, 113). Moreover, Liu et al reported that a significantly greater proportion of patients who presented with an NLR >3.13 progressed to critical illness when compared to those with an NLR <3.13, highlighting the potential of NLR as a marker by which to stratify patients based on their risk of disease progression (112). Thus, an early assessment of NLR may help identify patients that are likely to require more intensive monitoring and clinical care.

#### Neutrophil Phenotype

Until recently, neutrophils were considered a homogeneous population of terminally differentiated short-lived cells whose only role in host defence was the detection and elimination of pathogens. However, over the past decade it has been shown, under homeostatic and inflammatory conditions, that neutrophils are in fact a diverse cell population that exhibit phenotypic and functional heterogeneity (114, 115). Currently, it is unclear as to whether this heterogeneity is borne from the presence of distinct neutrophil “subsets”, or simply reflects differences in cell maturity and/or activation status (116). Indeed, whilst it is accepted that neutrophils possess pro-inflammatory, immunoregulatory and immune suppressive properties (114, 115), assigning these features to distinct subpopulations is difficult due to a lack of defined markers and the contrasting functions reported for these populations across different disease settings (116). In the context of systemic inflammation, two neutrophil populations are receiving significant attention. The first, termed low-density neutrophils (LDNs), are a heterogeneous group of immature and activated mature cells, whose functional capacities vary depending on the disease context. For example, in cancer, LDNs display T cell suppressive activity, and are often referred to as granulocytic myeloid-derived suppressor cells (114, 116, 117). Conversely, in the settings of autoimmunity (e.g. systemic lupus erythematosus) and infection, LDNs have been reported to exhibit a pro-inflammatory phenotype, as evidenced by increased type 1 IFN production, ROS generation, degranulation and an activated surface phenotype (118, 119). With a propensity for enhanced cytokine production and spontaneous NET generation, low density granulocytes (LDGs) are a second “distinct” class of neutrophils, whose functional hyperactivity has been extensively studied in the context of autoimmunity (114, 120, 121). Using a range of analytical approaches that have included single cell RNA-seq (scRNA-seq), high-dimensional flow cytometry and mass cytometry, a number of studies have reported marked differences in the composition of the circulating neutrophil pool between COVID-19 patients of differing disease severities (18, 29, 48, 49, 122–125). Supporting the idea that distinct innate immune responses underlie the different clinical trajectories of COVID-19, an accumulation of LDNs is emerging as a signature of severe disease (18, 21).

Defined by a broad range of surface phenotypes that have included CD16^low/high^CD10^-^, CD15^+^CD10^-^CD66b^+^, CD10^-^CD64^+^, CD10^-^, CD10^-^CD16^-^CD66b^+^ or CD10^low^CD101^+/-^, significantly increased frequencies and absolute numbers of immature neutrophils have been detected in patients with COVID-19 when compared to HCs (48, 49, 122, 126–128). In depth characterisation of these cells has identified a CD16^Int^CD44^Low^CD11b^Int^ subpopulation that possess characteristics reminiscent of LDGs. Referred to as low-density inflammatory band cells (LDIBs), these cells, whose circulating frequencies are significantly higher in patients with severe COVID-19 when compared to mild cases (29), exhibit spontaneous NET formation and LPS-induced pro-inflammatory cytokine production, with the generation of IL-6 and TNF-α significantly higher for LDIBs of severe COVID-19 cases when compared to mild patients (29). Further demonstrating severity-associated differences in LDNs, Combadiere et al detected an increased frequency of CD123^+^ and LOX1^+^ immature neutrophils in ICU patients when compared to non-ICU cases (125). Interestingly, suggesting that heterogeneity also exists within the neutrophil compartment of patients with severe disease, the authors reported an increased frequency of CD123^+^ and LOX1^+^ immature neutrophils in COVID-19 ICU patients that they classified as having more severe disease (defined as a sequential organ failure assessment (SOFA) score >8) when compared to ICU patients with a SOFA score <8 (125). In terms of longitudinal sampling, studies have reported a significant reduction in LDN frequencies over the course of COVID-19 (122), with convalescent patients presenting with absolute numbers of CD16^low/high^CD10^-^ neutrophils that are significantly lower than those in patients with active disease and comparable to those recorded in HCs (126). Based on these observations, the presence of circulating LDNs is considered an early marker of SARS-CoV-2 infection (126).

ScRNA-seq of whole blood, peripheral blood mononuclear cells (PBMCs) and purified neutrophils isolated from COVID-19 patients of differing disease severities has revealed unique transcriptomic signatures for neutrophils of patients with severe disease (17, 18, 21, 124). Schulte-Schrepping et al identified two distinct subpopulations of LDNs in PBMC samples of patients with severe disease (18), with detailed analysis revealing eight distinct cell clusters consisting of pro-, pre- and mature neutrophils that expressed genes involved in NET formation (e.g. MPO, ELANE, PRTN3 and PADI4) and the suppression of adaptive immune responses (e.g. CD274 and ARG1) (18). In line with these observations, Aschenbrenner et al performed transcriptomic profiling on whole blood samples and purified neutrophils from mild and severe COVID-19 patients and showed signatures associated with neutrophil activation (e.g. CD177, PADI4, ELANE and MPO) and immune suppression (e.g. ARG1 and CD274) were enriched in patients with severe disease (17). Interestingly, the authors also showed that COVID-19 patients presented with combinations of gene clusters that were not detected in the whole blood transcriptome of patients with other infectious diseases or inflammatory-associated conditions (17). Moreover, mass cytometry of whole blood samples has shown that in contrast to the immune response of patients with SARS-CoV-2 negative flu-like illness, which was characterised by the presence of highly activated mature neutrophils, an accumulation of activated immature neutrophils with immune suppressive properties defined the granulocyte response of patients with severe COVID-19 (18). Taken together, these studies suggest that SARS-CoV-2 infection induces a distinct neutrophil response that is enriched with features of immaturity, activation and suppression.

The presence of immature neutrophils in the circulation of COVID-19 patients suggests SARS-CoV-2 infection triggers emergency granulopoiesis, the premature mobilisation of neutrophil precursors from the bone marrow. Emergency granulopoiesis has previously been reported in the setting of trauma and sepsis, where systemic inflammation is considered a key factor in driving the release of neutrophil progenitors from the bone marrow (129, 130). Suggesting that this may also be the case in COVID-19, significant positive associations have been reported between plasma concentrations of IL-6 and the frequency/absolute number of CD10^-^CD64^+^Lox1^+^ and CD16^low/high^CD10^-^ neutrophils as well as CD16^Int^CD44^Low^CD11b^Int^ LDIBs (29, 125, 126). Moreover, circulating concentrations of G-CSF, which is considered a key mediator of emergency granulopoiesis (129, 131), are significantly higher in COVID-19 patients when compared to HCs, with those with severe disease exhibiting higher concentrations than moderate cases (5).

#### Degranulation

Located in the membranes of primary, secondary and tertiary granules respectively, increased expression of CD63, CD11b or CD66b on the neutrophil cell surface is considered a surrogate marker of degranulation. Studies that have examined neutrophil degranulation via these changes in surface phenotype have reported conflicting results, with some groups describing a SARS-CoV-2-induced increase in the expression of CD63 and CD66b (26, 122, 128) and others reporting no difference in the expression of CD11b or CD66b between neutrophils isolated from COVID-19 patients of differing disease severities and HCs (122, 132). In contrast, studies that have measured the circulating concentrations of granule-derived proteins released by activated neutrophils [e.g. neutrophil elastase (NE), myeloperoxidase (MPO) and matrix metalloproteinase-9 (MMP-9)] have consistently reported robust neutrophil degranulation in response to SARS-CoV-2 infection.

NE is a serine protease stored within the primary granules of neutrophils. Compared to the levels recorded for HCs, both the concentration and activity of NE has been found to be significantly higher in blood samples from severe COVID-19 patients with ARDS and/or patients with disease severities ranging from mild to fatal (26, 122, 133, 134). When released into plasma, NE is rapidly inactivated by endogenous protease inhibitors. Interestingly, sera from COVID-19 patients has been shown to significantly inhibit the activity of exogenous NE in *in vitro* assays, suggesting that the increased activity of NE in the blood of COVID-19 patients reflects a degree of resistance of the protease to its endogenous inhibitors (26). On this note, NE is protected from plasma-mediated neutralisation when bound to DNA (135, 136). Thus, the significantly higher number of DNA-NE complexes that have been measured in SARS-CoV-2 infected patients offers a potential mechanistic explanation for the increased activity of NE in COVID-19 blood samples (26). In addition to causing bystander tissue damage that may contribute to the onset of acute lung injury (ALI) in COVID-19 patients, an elevation in the concentration and activity of NE may enhance SARS-CoV-2 infectivity (137, 138). SARS-CoV-2 enters cells directly from the cell surface when its surface expressed spike glycoprotein is cleaved by host proteases such as transmembrane serine protease-2 (TMPRSS-2), a modification that enables the virus to bind to its surface ACE2 (80). NE has been shown to cleave the spike glycoprotein of SARS-CoV viruses (139, 140). Thus, one can envisage a vicious cycle in patients with severe COVID-19 whereby SARS-CoV-2-induced activation of neutrophils triggers the release of NE, whose subsequent cleavage of the spike glycoprotein facilitates enhanced viral entry, which results in increased viral infectivity and disease pathogenesis (138).

When compared to HCs and patients with mild-to-moderate disease, significantly higher concentrations of MPO have been measured in blood samples acquired from severe COVID-19 patients (128, 141). Suggesting a relationship between increased neutrophil activation and disease severity, Ueland and colleagues reported a negative correlation between plasma MPO levels and Pa02/Fi02 (P/F) ratio, a marker of respiratory function for which low values indicate respiratory distress/failure (141). In a recent review article, in which it was suggested that MPO release is a consequence of the cytokine storm that is induced by SARS-CoV-2 infection, Goud et al addressed a potential role for elevated MPO levels in driving the clinical deterioration that precedes the onset of severe COVID-19 (142). Mechanistically, the authors suggested that by catalysing the generation of the oxidant hypochlorous acid and consuming nitric oxide, MPO may contribute to COVID-19 pathology by promoting oxidative stress, vasoconstriction, tissue injury and cell death (142).

Positively associated with neutrophil number, plasma concentrations of MMP-9 are significantly elevated in SARS-CoV-2 infected patients (105). A protease involved in the degradation of the extracellular matrix (ECM), activity of MMP-9 is increased when it is bound to the protein neutrophil gelatinase-associated lipocalin (NGAL), whilst its activity is negatively regulated by tissue inhibitor of metalloproteinase-1 (TIMP-1) (143, 144). Relative to HCs, significantly elevated concentrations of MMP-9:NGAL heterodimers and an increased MMP-9:TIMP-1 ratio have been detected in COVID-19 patients (105). Pointing towards enhanced plasma MMP-9 activity, which has previously been linked to the development of ARDS in critically-ill patients (145), these findings suggest that an increase in MMP-9-mediated proteolysis may, by promoting lung injury, contribute to the onset of severe COVID-19 (105). Supporting this idea, Ueland et al reported a significant negative correlation between plasma MMP-9 levels and P/F ratio in 39 hospitalised COVID-19 patients, with those who developed respiratory failure presenting at two study time-points (days 0-2 and 3-5 post-hospitalisation) with significantly higher circulating concentrations of MMP-9 than patients who did not experience respiratory failure (146). Interestingly, in a recent network-based systems biology study that set out to identify the molecular targets of drugs that have been proposed as potential candidates for the treatment of COVID-19, MMP-9 emerged as an interacting partner of both chloroquine and melatonin (147). Whilst current evidence suggests chloroquine treatment does not improve clinical outcomes in patients with COVID-19 (148), high dose melatonin has shown promise as an adjuvant therapy, with evidence of potential clinical benefit in hospitalised patients with COVID-19 pneumonia (149). Whilst the mechanism of action of melatonin is likely to be multi-factorial (150), its ability to significantly inhibit MMP-9 activity and expression (151) suggests that its clinical benefit could arise, at least in part, from a reduction in MMP-9 mediated inflammatory responses and lung damage.

#### NET Formation

NETs are an extracellular defence mechanism involved in the capture, neutralisation and elimination of invading pathogens (152). Renowned for their role in immune responses against bacteria and fungi (153, 154), data are now emerging demonstrating that NETs are also generated in response to viral challenge (155–158). Consisting of a DNA backbone decorated with histones and an array of granule-derived peptides and enzymes such as MPO, NE and gelatinase, NETs have significant cytotoxic and thrombotic potential (153). Thus, NETs have been proposed to represent “double-edged swords”, with their excessive or dysregulated production suggested to promote tissue damage, thrombotic complications and/or hyperinflammation, all of which offset their anti-microbial properties (159, 160). In the context of respiratory infections, aberrant NET formation has been associated with the development of ALI and ARDS (161–163), two conditions that, alongside multiple organ failure (MOF) are common secondary complications of severe COVID-19 (30, 164, 165). Thus, in an effort to establish whether NETs contribute to the pathogenesis of severe COVID-19, a number of studies have screened peripheral blood and tracheal aspirate samples from COVID-19 patients of differing disease severities for evidence of NET formation.

Concentrations of a range of NET-associated markers, including cell-free DNA (cfDNA), MPO/NE-DNA complexes and citrullinated histone H3 (CitH3), have all been shown to be significantly increased in the circulation and/or tracheal aspirates of patients with COVID-19 (26, 46, 65, 66, 105, 134, 166–170). As expected for a respiratory infection, NET levels are significantly higher in tracheal aspirates, bronchiolar lavage and BALF samples when compared to plasma (65, 66). In terms of disease severity, patients with severe disease present with significantly higher circulating levels of cfDNA, MPO-DNA complexes and CitH3 when compared to patients with mild to moderate COVID-19 (26, 105, 170), with one study showing a surge in plasma NET markers prior to ICU admission (170). Thus, in heterogeneous patient cohorts, plasma NET levels positively correlate with clinical severity scoring systems (65, 105, 170) and are negatively associated with the P/F ratio (65). Interestingly, concentrations of MPO-DNA complexes have been shown to be significantly higher in plasma samples from male patients when compared to their female counterparts (105), an observation worthy of further investigation given that male gender is a risk factor for ICU admission and death following SARS-CoV-2 infection (171). To date, few studies have measured NET generation longitudinally in COVID-19 patients, with those that have reporting conflicting observations (65, 66, 105). For example, Middleton et al measured comparable levels of MPO-DNA complexes in plasma samples obtained from convalescent patients and HCs (65), whereas Petito and co-workers detected similar concentrations of MPO-DNA complexes in plasma samples collected from patients during active infection and recovery (105). Thus, it remains to be determined whether patients who survive severe COVID-19 are at an increased long-term risk of NET-associated complications.

Treating neutrophils isolated from HCs with serum/plasma from COVID-19 patients triggers NET production *in vitro*, thereby demonstrating the presence of NET-inducing agents in the circulation of SARS-CoV-2 infected subjects (65, 91, 105, 167, 172, 173). Pro-inflammatory cytokines (e.g. IL-6, IL-8, G-CSF), the complement factor C5a, antigen-antibody complexes and platelet derived factors (e.g. platelet factor 4 (PF4) and RANTES), whose circulating levels are all increased in patients with severe COVID-19 (5, 65, 91, 167, 174, 175), have been proposed as potential mediators of NET formation (65, 91, 167, 173). Interestingly, adding to a growing list of viruses, which includes chikungunya virus and human immunodeficiency virus (156, 157), SARS-CoV-2 directly promotes NET release (46, 172). Dependent upon the activity of peptidylarginine deiminase 4 (PAD4), TMPRSS-2 and the expression of ACE2 (46), SARS-CoV-2 triggers a level of NET production comparable to that observed for neutrophils treated with the potent protein kinase C agonist phorbol 12-myristate 13-acetate (172). Via its involvement in the detection of single-stranded RNA viruses, the intracellular pathogen recognition receptor toll-like receptor (TLR)-7 has been implicated in SARS-CoV-2-induced NET formation (46), although this remains to be proven.

In the absence of exogenous stimulation, neutrophils isolated from COVID-19 patients exhibit enhanced NET generation *in vitro* (46, 65, 168, 175). Mechanistically, this increase in spontaneous NET formation may reflect SARS-CoV-2-induced changes in the composition of the circulating neutrophil pool, specifically the presence of hyperactive neutrophils and/or immature LDNs. Indeed, preliminary data from Parackova and colleagues has shown evidence of increased activation of p38 and c-Jun N-terminal kinase, two kinases important for NET production (176, 177), in resting neutrophils isolated from COVID-19 patients (122). Moreover, LDNs, whose circulating frequencies are increased in patients with severe disease (128), exhibit enhanced spontaneous NET formation when compared to mature neutrophils (121, 178).

#### Metabolism

Results of metabolomic and lipidomic profiling on plasma and serum samples from COVID-19 patients of varying disease severities suggest that metabolic dysregulation contributes to disease pathogenesis, with perturbations reported in such pathways as tryptophan metabolism, the tricarboxylic acid (TCA) cycle, mobilisation of polyunsaturated fatty acids and eicosanoid biosynthesis (32, 179–184). Alongside these changes in the systemic metabolome, data are accumulating that suggest SARS-CoV-2 infection promotes metabolomic reprogramming in neutrophils (128, 185) and PBMCs (181).

In a study that compared metabolic processes between neutrophils isolated from HCs and severe COVID-19 patients, McElvaney et al demonstrated a disease associated increase in the cytosolic levels of pyruvate kinase M2, an enzyme that catalyses the final step of glycolysis, and the nuclear expression of hypoxia-inducible factor-1α, a transcription factor that promotes the expression of glycolytic enzymes (185). A profile that would be predicted to enhance glycolytic flux, the authors found cytosolic lactate levels and the lactate:pyruvate ratio were significantly higher in neutrophils of COVID-19 patients, suggesting a SARS-CoV-2-induced shift of neutrophil metabolism towards glycolysis (185). Critical for neutrophil anti-microbial responses, glycolysis and lactate generation are essential steps in the formation of NETs (186, 187). Thus, this elevated glycolytic activity of COVID-19 neutrophils could be a mechanistic explanation for the aforementioned increase in spontaneous *ex vivo* NET generation by neutrophils isolated from patients with severe disease (46, 65, 168, 175). However, in an independent study, Reyes et al provided evidence of reduced glycolytic flux and a switch to oxidative metabolism in neutrophils isolated from COVID-19 patients with ARDS (128). Reflecting the infancy of this field of research, these conflicting observations are likely to be a consequence of small cohort sizes (n=3-8) and differences in methodological approach. Thus, further studies enrolling a larger number of patients with varying disease severities are required to fully understand how SARS-CoV-2 infection impacts upon neutrophil metabolism.

### Pulmonary Neutrophil Response

As a lower respiratory tract infection, the greatest insight into the immune response to SARS-CoV-2 has been provided by studies that have directly examined lung tissue, via histological staining of post-mortem COVID-19 lung samples, or analysed the pulmonary microenvironment via the acquisition of BALF. Although hampered by small sample sizes and the decision to often use samples collected from HCs as a reference cohort rather than patients with non-COVID-19 respiratory infections, current data suggest that robust chemokine production and dysregulated myeloid responses, which include the infiltration and activation of neutrophils, characterise the pulmonary immune responses of patients who develop severe COVID-19.

#### Analysis of BALF

Our current understanding of how SARS-CoV-2 infection influences the neutrophil pool within the lung is based almost entirely upon the findings of studies that have performed scRNA-seq on BALF samples obtained from COVID-19 patients of different disease severities (48, 78, 97, 188–193). Pathway analysis of the differentially expressed genes that have been identified between the BALF transcriptomes of COVID-19 patients and HCs has revealed chemokine signalling to be one of the most up-regulated pathways in SARS-CoV-2 infected subjects (189). In terms of specific transcripts, the expression levels of genes relating to neutrophil chemotaxis such as CXCL1, CXCL2, CXCL6, CXCL8 and CCL2 have consistently been found to be up-regulated in COVID-19 patients (189–192). Moreover, suggesting that SARS-CoV-2 triggers a robust neutrophilic response, the up-regulation of neutrophil chemokine associated genes in COVID-19 patients is significantly greater than that reported in BALF from patients with viral or non-viral community-acquired pneumonia (189). A combination of *ex vivo* data (188, 193) and results from *in vitro* cell culture systems (191, 194, 195) suggest that this chemokine signature of SARS-CoV-2 infection is expressed predominantly within lung macrophages and both alveolar and bronchial epithelial cells. Interestingly, suggesting a breakdown in regulatory negative feedback loops, infection of primary human lung epithelial cells *in vitro* with SARS-CoV-2 was found to result in down-regulation of the gene encoding for CXCL14 (195). A negative regulator of immune cell recruitment, it has been suggested that, if replicated *in vivo*, reduced expression of CXCL14 would result in enhanced recruitment of neutrophils to the lung (195). In line with these transcriptomic data, increased concentrations of IL-8 have been measured in the BALF of patients with severe COVID-19 when compared to those with moderate disease (188).

As expected for a microenvironment enriched in neutrophil chemoattractants, neutrophil associated transcripts are more abundant in the BALF samples of severe COVID-19 patients when compared to those of HCs (48, 189, 190), subjects with mild disease (48, 190, 196) or patients with non SARS-CoV-2-induced pneumonia (78, 189). This enrichment in neutrophil signatures has been shown to be independent of lung injury, age, gender and underlying co-morbidities (78), suggesting that rather than simply representing a marker of severe lung inflammation, neutrophils contribute to the immunopathology that underpins the onset of COVID-19 pneumonia (78). Akin to the findings reported in whole blood (17, 18, 21), heterogeneity has also been reported in the pulmonary neutrophil pool (48, 78). Wauters et al clustered BALF residing neutrophils into five distinct phenotypes and found the frequency of both “progenitor” and “inflammatory mature” neutrophils were significantly higher in samples from mild/critical COVID-19 patients when compared to subjects with non-COVID-19 pneumonia (78). Interestingly, phenotypic profiling of immature CD16^Int^CD44^Low^CD11b^Int^ LDIBs, whose circulating frequencies are increased in severe COVID-19 patients, has shown that this subpopulation exhibits decreased surface expression of CD44 (29). As low CD44 expression is associated with increased neutrophil migration to the lungs (197), it may be that pulmonary infiltration of LDIBs contributes to the “progenitor” phenotype of neutrophils detected in the BALF samples of critical COVID-19 patients (78).

In terms of functional responses, gene sets related to neutrophil degranulation and NETs are up-regulated in lung/BALF samples of COVID-19 patients (97). Interaction network analysis has shown these NET associated genes interact with negative regulators of T and natural killer cell function as well as genes linked to ROS formation (97). Thus, it has been suggested that in addition to promoting direct tissue injury, NET formation within the lungs of COVID-19 patients may hasten viral replication and disease pathogenesis by impairing host anti-viral immune responses (97).

#### Analysis of Lung Tissue

Histopathological staining of post-mortem lung tissue has demonstrated intense neutrophilic infiltrate within the alveolar spaces and/or pulmonary vessels of patients with severe COVID-19 (37, 46, 65–68). Furthermore, and in agreement with data obtained from total RNA-seq (198), NETs have been identified throughout the lungs of fatal COVID-19 cases. Defined as extracellular areas of DNA staining positive for MPO and CitH3, NETs have been detected in the airway, interstitial, alveolar and vascular compartments of COVID-19 lungs (26, 46, 65, 66, 199). Suggesting that NET formation occurs only in the lungs, Radermecker et al failed to detect evidence of NETs in tissue sections obtained from the liver, pancreas, kidney or heart of SARS-CoV-2 infected patients (199). However, in contrast, Leppkes and colleagues showed the presence of aggregated NETs in both kidney and liver tissue samples, leading them to propose that widespread NET generation may contribute to multi-organ damage in cases of severe COVID-19 (26). To address these conflicting observations, and establish whether NET formation in peripheral organs is indeed a feature of severe disease, additional studies with larger sample sizes are required.

Although SARS-CoV-2 can directly induce NET formation (46, 172), only a small number of viral transcripts have been detected in post-mortem lung samples (198), suggesting that other agonists may be triggering pulmonary NET formation in severe COVID-19. Potential candidates that have been proposed thus far include PF4, whose gene expression is increased ~5.5 fold in lung tissue of fatal COVID-19 cases (198), and an array of NET-inducing cytokines and chemokines such as IL-6, IL-8, TNF-α, IL-1β and CCL20 whose expression levels are up-regulated in COVID-19 BALF samples and/or human lung epithelial cells infected with SARS-CoV-2 (188, 189, 194, 195). The low viral burden present in post-mortem lung tissue has led to the suggestion that rather than an ongoing active viral infection, tissue injury and inflammation arising from dysregulated and overexuberant host immune responses is responsible for patient death (198). Due to their cytotoxic and pro-coagulant nature (159, 160), aberrant NET formation may be one factor contributing to the diffuse alveolar damage and interstitial lesions that have been detected in the lung samples of fatal COVID-19 cases (37, 46, 199, 200).

In an elegant study that investigated the early innate immune response to SARS-CoV-2 infection, Guo et al found a greater number of genes related to “cellular responses to lipopolysaccharide (LPS)” and “neutrophil chemotaxis” were up-regulated in lung tissue samples obtained from rhesus macaques and mice at days 3 and 5 post-infection when compared to genes involved in anti-viral immunity (201). Not observed in mice infected with other RNA or DNA viruses, SARS-CoV-2 triggered the infiltration of immature CD45^+^11b^+^Ly6G^variable^ aberrant neutrophils into the lung, which was associated with fatal outcome (201). Mechanistically, this anti-bacterial response to viral challenge was attributed to robust induction of S100A8, an alarmin that upon binding to S100A9 forms calprotectin, a damage associated molecular pattern (DAMP) that promotes neutrophil chemotaxis and activates TLR4, the pathogen recognition receptor for LPS (201–203). Indeed, associated with increased survival rates, significantly fewer immature neutrophils and reduced viral loads were recovered from lung samples of SARS-CoV-2 infected mice treated with paquinimod, an inhibitor of S100A8/S100A9 signalling (201). In addition to the lungs, these aberrant immature neutrophils, which were suggested to be granulocytic myeloid-derived suppressor cells, were also detected in the blood and bone marrow of SARS-CoV-2 infected mice, demonstrating systemic dysregulation of the neutrophil pool (201). Interestingly, relative to HCs, increased expression of S100A8 has also been detected in lung tissue samples from patients with severe COVID-19 (198, 201), who, when compared to moderate cases, also present with significantly higher circulating concentrations of calprotectin (48, 204, 205). Thus, based on the observations of Guo et al (201), and data that has shown calprotectin stimulates granulopoiesis (206), neutrophil chemotaxis (202) and NET formation (207), the systemic and pulmonary elevation that occurs in the levels of this DAMP could promote the premature mobilisation and infiltration of immature neutrophils into the lungs, where they contribute to tissue injury and pulmonary failure.

## Neutrophil Tolerance in Severe COVID-19

With an incidence rate of 13.5-56%, hospital-acquired infections are emerging as a common secondary complication amongst COVID-19 patients (175, 208–210). More frequently observed in those with severe disease (209), secondary bacterial and fungal superinfections have been shown to be significantly associated with an increased hospital and ICU length of stay, an increased need for mechanical ventilation and a lower 28-day ventilator-free survival rate (208). This susceptibility to fungal and bacterial infections suggests that neutrophils of COVID-19 patients exhibit impaired anti-microbial functions upon secondary challenge. Supporting this idea of functional tolerance/exhaustion, significantly reduced ROS production, degranulation and NET formation have been reported for neutrophils isolated from COVID-19 patients following *in vitro* stimulation with bacteria (e.g. *Staphylococcus aureus, Streptococcus pneumonia or Escherichia coli*) or bacterial products (LPS), all of which culminate in reduced bactericidal activity (18, 26, 65, 175).

Suggesting the presence of an inhibitory/tolerising factor in the circulation of COVID-19 patients, Shambat et al demonstrated that pre-treating neutrophils isolated from HCs with COVID-19 patient plasma resulted in significantly impaired functional responses upon bacterial challenge (175). Mechanistically, the authors suggested IL-4 and IL-10, two anti-inflammatory cytokines that inhibit neutrophil function (211, 212), and whose circulating concentrations are significantly increased in SARS-CoV-2 infected patients (5, 175), may be responsible for the plasma-driven reduction they observed in neutrophil activity (175). However, it is unlikely that these cytokines are wholly responsible. For instance, pre-treatment of HC neutrophils with the complement factor C5a, whose circulating concentrations are increased in severe COVID-19 patients (91, 174), has been shown to reduce both phagocytic and bactericidal activity (213, 214). Moreover, due to the inflammation-induced tissue damage that would occur in patients with severe COVID-19, we suggest that the release of mitochondrial-derived damage associated molecular patterns (mtDAMPs) may also contribute to the SARS-CoV-2-induced induction of neutrophil tolerance.

Passively released from damaged/necrotic tissue and secreted by activated immune cells, damage associated molecular patterns (DAMPs) are a heterogeneous collection of nuclear, cytosol and mitochondrial-derived proteins, lipids and DNA (215). Owing to their prokaryotic origins, mtDAMPs are potent immune activators, with their stimulation of neutrophils linked to the induction of systemic inflammatory responses and organ injury (216–218). However, in addition to immune activation, we and others have shown that mtDAMPs induce functional tolerance, with neutrophils pre-treated with mtDAMPs or mtDAMP mimetics exhibiting significantly impaired calcium mobilisation, chemotaxis and NET generation upon secondary stimulation with inflammatory or pharmacological agonists (216, 219–221). To date, whilst several studies have reported evidence of cellular injury and inflammatory cell death in patients with severe COVID-19 (23, 26, 222), few have directly screened blood samples for the presence of DAMPs. However, in those that have, significantly elevated levels of the nuclear-derived DAMPs high mobility group box-1 (HMGB-1) and extracellularly-secreted nicotinamide phosphoribosyl-transferase (eNAMPT), as well as mitochondrial-derived DNA (mtDNA) were detected in the plasma of severe COVID-19 patients when compared to HCs and/or mild to moderate COVID-19 cases (205, 223, 224). Given that N-formylated peptides, which we have shown are responsible for mediating mtDAMP-induced neutrophil tolerance (219), are released alongside mtDNA, then these preliminary data suggest that tolerising mtDAMPs are present in the circulation of patients with severe COVID-19. However, studies that quantify N-formylated peptides (e.g. NADH-ubiquinone oxidoreductase chain 6 (ND6)) in COVID-19 patient plasma and assess their relationship with *ex vivo* neutrophil function are needed to support our hypothesis that mtDAMPs contribute to SARS-CoV-2-induced neutrophil tolerance.

In the setting of non-COVID related ARDS, where elevated BALF concentrations of mtDNA and N-formylated peptides have been implicated in disease pathogenesis (225, 226), pharmacological manipulation of mtDAMP signalling has been suggested as a potential treatment for neutrophil-mediated lung injury (225). Similarly, with the aim of suppressing pulmonary inflammation and preventing the onset of severe disease, blocking DAMP signalling was recently proposed as a treatment for COVID-19 (223, 227). We believe that this approach, particularly when focussed upon mtDAMPs, may have the additional benefit of reducing the susceptibility of COVID-19 patients to superinfections by preventing mtDAMP-induced neutrophil tolerance. This viewpoint is shared by researchers in other fields of critical care who have proposed that mtDAMP exposure underlies the susceptibility of traumatically-injured patients to pulmonary infections and that blocking the formyl peptide receptor would, by restoring neutrophil function, enhance immune responses to bacterial challenge (221). Finally, whilst this discussion has focussed on neutrophil responses, it should be noted that monocytes of COVID-19 patients also exhibit impaired anti-microbial responses *ex vivo* and that pre-treating HC monocytes with COVID-19 patient plasma significantly reduced their bactericidal activity (175). Given that exposure to mtDAMPs also induces functional tolerance in monocytes (228), then it is reasonable to speculate that targeting mtDAMP signalling may restore the effector functions of two key innate immune cells, thereby reducing the susceptibility of COVID-19 patients to hospital-acquired superinfections.

## Are Neutrophils a Potential Therapeutic Target for the Treatment of COVID-19?

If a granulocytic signature characterised by immature, hyperactive and pro-inflammatory neutrophils is a feature of the dysregulated peripheral and pulmonary immune responses that contribute to the transition from mild to severe COVID-19 (17–21), then *does the neutrophil represent a potential therapeutic target for the treatment of hospitalised COVID-19 patients?* This question has been raised in a number of previous perspective and review articles, where an approach that targets specific signalling pathways and/or neutrophil products (e.g. NETs, ROS or proteases) appears to be favoured over strategies that would result in the complete suppression of neutrophil function (36, 37, 137, 138, 191, 229–231). Indeed, given the abovementioned susceptibility of hospitalised COVID-19 patients to bacterial and fungal superinfections (175, 208–210), a therapeutic strategy that suppresses neutrophil hyperactivity whilst preserving anti-microbial function would be best placed to reduce neutrophil-mediated bystander tissue damage without increasing the risk of secondary infections (231). In this section, we consider whether targeting NETs or suppressing the release of immature neutrophils from the bone marrow represent potential therapeutic strategies for the treatment of COVID-19.

### NETs

To date, discussions regarding the potential use of anti-neutrophil therapies for the treatment of COVID-19 have centred on targeting NETs, whose enhanced generation is likely to contribute to both the pulmonary and extrapulmonary manifestations of severe COVID-19 (231–233). Implicating NETs in the clotting associated complications that have been suggested to account for more than 70% of COVID-19 deaths (234), significantly higher plasma levels of cfDNA, MPO-DNA complexes and CitH3 have been detected in patients who developed thrombotic events when compared to those who did not (105, 170). In addition, excessive NET formation in the lungs has been shown to result in vascular occlusion and disturbed microcirculation (26). Through their ability to induce macrophages to secrete IL-1β (235), which is itself a stimulus for NET formation (236, 237), the presence of NETs within the lungs could foster a self-perpetuating and deleterious positive feedback loop that, by promoting a state of hyperinflammation, would accelerate the formation of microthrombi and the onset of respiratory failure (37). Moreover, with recent evidence demonstrating that NET bound NE inhibits efferocytosis by cleaving the integrins a_v_β_3_ and a_v_β_5_ from the surface of macrophages (238), increased NET formation may also delay the resolution phase of pulmonary immune responses in patients with severe COVID-19. Thus, via their potential involvement in facilitating immunothrombosis, pulmonary and systemic hyperinflammation and respiratory failure, NETs represent an ideal therapeutic target for treating a number of the complications experienced by severe COVID-19 patients. Potentially achievable via the repurposing of existing therapies, two strategies currently under consideration for alleviating NET-associated pathologies in SARS-CoV-2 infected patients are to (i) suppress NET formation or (ii) enhance NET breakdown.

#### Suppression of NET Formation

Promoting chromatin decondensation via the citrullination of histone H3, PAD4 is a major regulator of NET formation (239). Having shown promise in murine models of infection and inflammation, where inhibition of PAD4 resulted in reduced NET-associated lung injury (162, 240), systemic or pulmonary administration of PAD4 inhibitors has been proposed as a potential treatment for severe COVID-19 (36). Current data that would support such an approach are the results of studies that have shown *in vitro* treatment with Cl-amidine, an irreversible pan-PAD inhibitor, significantly reduced SARS-CoV-2-induced NET production by HC neutrophils (46) as well as the enhanced spontaneous NET generation of neutrophils isolated from COVID-19 patients (172).

Treating HC neutrophils *in vitro* with COVID-19 patient plasma/serum induces NET formation (65, 91, 105, 167, 172, 173). To date, C5a and antigen-antibody complexes have been proposed to be some of the inflammatory agonists that trigger this response (91, 173). If correct, then targeting these mediators directly or blocking their signalling pathways may represent another therapeutic approach by which to suppress NET production. In this area of research, Strich et al recently demonstrated that via inhibition of spleen tyrosine kinase (SYK), which signals downstream of FCγRIIA receptors, pre-treating neutrophils with R406, the metabolically active component of the food and drug administration (FDA)-approved drug fostamatinib, significantly reduced NET formation by HC neutrophils stimulated with COVID-19 patient plasma (173). Hypothesising that a reduction in NET generation may be one mechanism by which inhibition of SYK could improve the outcome of patients with COVID-19, a phase II, randomised, double-blind, placebo controlled trial of fostamatinib for the treatment of hospitalised COVID-19 patients is currently underway (ClinicalTrials.gov identifier: NCT04579393). Another drug under investigation in ongoing COVID-19 clinical trials is eculizumab (ClinicalTrials.gov identifiers: NCT04346797, NCT04355494, NCT04288713). A humanized IgG monoclonal antibody that inhibits the cleavage of the complement protein C5, eculizumab treatment has been shown to significantly reduce neutrophil activation in patients with a history of thrombosis (241, 242), which in one study was associated with a decline in circulating markers of NETs (242). Interestingly, clinical recovery following eculizumab treatment was recently reported in a case series of four COVID-19 ICU patients with severe pneumonia or ARDS, with reduced neutrophil activation and infiltration into the lungs proposed as potential mechanistic explanations (243). Follow-up studies that measure markers of neutrophil activation and NET generation in plasma and/or BALF samples of COVID-19 patients treated with eculizumab would help address this hypothesis. Alongside these potential therapies, inhibition of the abovementioned IL-1β-NET positive feedback loop could be achieved by treatment with anakinra. A human IL-1 receptor antagonist, anakinra is approved for use in patients with rheumatoid arthritis and has been shown to significantly reduce NET formation *in vitro* (236). Furthermore, in a murine model of airway inflammation, anakinra treatment resulted in a significant decrease in the BALF levels of the DAMP HMGB-1 (244). An inducer of NET formation (245, 246), significantly higher concentrations of HMGB-1 have been reported in severe COVID-19 patients when compared to both HCs and mild/moderate cases (205). Thus, via a number of mechanisms, antagonism of the IL-1 receptor may reduce the NET burden of COVID-19 patients, a hypothesis that can be tested by studying the results of an ongoing clinical trial that is measuring DNA-MPO complexes in serial blood samples acquired from severe COVID-19 patients receiving anakinra treatment (ClinicalTrials.gov identifiers: NCT04594356).

Compared to subjects who received standard anti-viral and steroid therapy, an open label study of 31 severe COVID-19 patients reported improved clinical indices and remission rates for patients co-treated with the FDA-approved drug dipyridamole (247). An anti-thrombotic medication, dipyridamole was recently shown to suppress NET formation *in vitro* via activation of the adenosine A_2A_ receptor (248). Based on these observations, it was suggested that a randomised trial that would assess the impact of dipyridamole treatment on NET formation and its relationship with clinical outcome in COVID-19 patients would be of interest (167). Since this proposal, Petito et al have shown that pre-treatment of HC neutrophils with dipyridamole failed to inhibit NET formation triggered by COVID-19 patient plasma (105). Thus, whilst dipyridamole may not suppress NET generation in SARS-CoV-2 infected subjects, the idea of activating signalling pathways that negatively regulate NET formation is worth pursuing as a therapy for COVID-19. Under consideration as a strategy for reducing NET generation in other inflammatory conditions is treatment with prostaglandin E2 (PGE_2_), which potently suppresses NET formation by increasing intracellular levels of cyclic adenosine monophosphate (249, 250). However, due to the dysregulated lipid profiles of COVID-19 patients (32) and the potent immunomodulatory properties of PGE_2_, which extend beyond the inhibition of NET formation, more studies are required to address the practicalities of such an approach in SARS-CoV-2-infected patients.

Whilst the idea of blocking NET formation is gaining traction as a potential therapy for the treatment of severe COVID-19 (36, 37, 173), we feel it is important to highlight a potential caveat of such an approach: reduced anti-microbial immunity. Although deleterious when generated in excess, NETs play an important role in the entrapment, neutralisation and eradication of bacterial and fungal pathogens (153, 154). Thus, the use of PAD4 inhibitors for example, may increase the susceptibility of severe COVID-19 patients to secondary infections. Indeed, whilst it has been shown that the ability of neutrophils isolated from PAD4^-/-^ mice to phagocytose and generate ROS is comparable to that of neutrophils from wild type littermates (251), PAD4^-/-^ mice showed impaired bacterial clearance in a model of pneumonia-induced ALI (162). Thus, the reported benefit of reduced NET-mediated lung injury in PAD4^-/-^ mice was offset by an increased bacterial burden (162). Such factors as dosage, timing and duration of therapy may therefore prove to be critical in determining the outcome of any strategy that attempts to eradicate NET formation for the treatment of COVID-19.

#### Degradation of NETs

Responsible for the breakdown of 90% of circulating cfDNA, deoxyribonuclease-1 (DNase-1) is the predominant endonuclease in bodily fluids (252). Relative to HCs, patients with severe, but not mild, COVID-19 exhibit significantly reduced circulating levels of this endonuclease (166), whose plasma concentrations have also been shown to be significantly lower in COVID-19 non-survivors when compared to survivors (169). In terms of function, significantly impaired total DNase activity has been measured in serum samples acquired from COVID-19 patients with mild and severe disease (233). Thus, the associations that have been reported between elevated NET markers, clinical deterioration and mortality could be attributable in part to a severity-associated impairment in DNase-1 mediated clearance of cfDNA (65, 105, 167, 169).

Used in the treatment of cystic fibrosis to aid in mucociliary clearance, dornase alfa is an FDA-approved recombinant human DNase-1 that digests extracellular DNA. Designed in part on the idea that exaggerated NET formation contributes to pulmonary hyperinflammation, a prospective randomised multi-centre clinical trial entitled COVIDornase is underway to investigate the effect of twice daily aerosolized intra-tracheal administration of dornase alfa on the severity and progression of ARDS in critically ill ICU COVID-19 patients (253). Potential benefits that have been proposed may arise from DNase-1 therapy in COVID-19 patients are enhanced clearance of mucous secretions, improved ventilation and a reduced risk of secondary infections (37, 253). However, whilst a promising therapy, it is important to highlight some potential barriers that we feel need to be considered and overcome if DNase-1 treatment is to be of clinical benefit for patients with severe COVID-19. These are:

1. *Timing of treatment* – In a murine model of sepsis, Mai et al demonstrated that despite reducing plasma levels of cfDNA, early, but not late, administration of DNase resulted in increased plasma concentrations of IL-6, enhanced inflammatory cell infiltration into the lungs and multi-organ damage (254). In the context of COVID-19, particularly patients with secondary infections, inappropriate timing of DNase therapy could therefore potentially exacerbate the pre-existing states of systemic and pulmonary hyperinflammation.
2. *Substrate* – Relative to HCs, both the circulating concentration and pulmonary expression of PF4 have been shown to be increased in COVID-19 patients (65, 198). Under flow conditions *in vitro*, PF4 binds directly to NETs resulting in their compaction, a morphological change that confers resistance to degradation by DNase-1 (255). Thus, the efficiency of DNase-1-mediated breakdown of NETs could be reduced in COVID-19 patients.
3. *Microenvironment* – Released into the circulation as a consequence of cell damage or death, actin is a negative regulator of DNase-1 activity (256, 257). Responsible for the clearance of circulating actin is the extracellular actin scavenger system (EASS), which comprises of two plasma proteins: vitamin D binding protein (VDBP) and gelsolin (GSN) (258). In the setting of thermal injury, we have recently demonstrated dysregulation of the EASS, where reduced circulating concentrations of GSN and VDBP were associated with impaired serum DNase activity and an accumulation of cfDNA (259). A similar scenario is emerging in COVID-19, where a significant reduction in total serum DNase activity (233) is accompanied by increased and decreased circulating concentrations of actin and GSN respectively (179, 260, 261), with GSN levels negatively associated with disease severity (260). Thus, we hypothesise that exogenous DNase-1 would be administered into an environment rich in circulating actin, which would result in the formation of DNase1-actin complexes and an impairment in endonuclease activity. A potential approach that could circumvent this issue is the use of AIR DNase™, an actin-resistant endonuclease whose safety, tolerability and efficacy has been tested in phase 2 trials of cystic fibrosis patients (ClinicalTrials.gov identifier: NCT02722122). Alternatively, a reduction in NET levels could be achieved by replenishing the EASS via supplementation with recombinant human plasma GSN (Rhu-pGSN), an approach that could restore the activity of endogenous DNase by promoting its dissociation from actin (262). A phase 2, randomised, double-blind, placebo-controlled trial of Rhu-pGSN in severe COVID-19 patients with pneumonia that will measure inflammatory biomarkers is currently underway (ClinicalTrials.gov identifier: NCT04358406).
4. *Generation of toxic degradation products* – As an endonuclease, DNase-1 is unable to degrade NET-associated proteins. Indeed, in a murine model of bloodstream infection, histones and NE remained attached to the walls of liver sinusoids following intravenous DNase treatment (263). Combined with the results of an *in vitro* study that demonstrated NET degradation by DNase-1 resulted in enhanced NE activity (264), then it is conceivable that DNase-1 treatment in COVID-19 patients could potentiate organ damage by exposing endothelial cells to cytotoxic histones (265) and releasing proteolytically active NE from NETs. A combined therapeutic approach of DNase-1 with either histone blocking antibodies (266), small molecular inhibitors of NE (e.g. sivelestat or alvelestat) or heparin could help eradicate any potential bystander tissue damage that would arise from DNase-1-mediated digestion of NETs. Of these strategies, co-therapy with heparin may be particularly beneficial given that this anti-coagulant has been shown to (i) reduce histone cytotoxicity towards endothelial cells (267), (ii) accelerate DNase-1-mediated breakdown of NETs (26) and (iii) reduce NET formation triggered by IL-8 and HMGB-1 (268), two pro-inflammatory agonists whose circulating concentrations are increased in patients with severe COVID-19 (5, 205).

### Immature Neutrophils

Calculated by subtracting the fraction of mature neutrophils from the sum of MPO-reactive cells, the delta neutrophil index (DNI) reflects the circulating fraction of immature neutrophils. In a study of 388 ICU patients, Birben et al found that the DNI, calculated at the time of hospital/ICU presentation, was significantly higher in COVID-19 patients who died within the first 30 days of ICU admission when compared to those who survived (269). Mechanistically, this relationship between increased neutrophil immaturity and poor outcome may be attributable to the pro-inflammatory nature of immature neutrophils, whose propensity for enhanced NET formation and cytokine production could contribute to systemic inflammation and tissue damage.

Compared to HCs, COVID-19 ICU patients exhibit significantly increased plasma concentrations of GM-CSF (5). Systemic elevation of this growth factor triggers the premature release of immature myeloid cells from the bone marrow. Thus, *could inhibiting the activity of GM-CSF improve the outcome of COVID-19 patients by suppressing the early mobilisation of pro-inflammatory immature neutrophils into the circulation?* Currently, a series of randomised placebo-controlled clinical trials are assessing the efficacy and safety of GM-CSF based therapies in patients with severe COVID-19 (270). Using a range of GM-CSF targeting monoclonal antibodies that include lenzilumab, otilimab, namilumab and gimsilumab, these trials are investigating the impact of GM-CSF neutralisation on such clinical outcomes as mortality, hospital/ICU LOS and ventilator-free days (270). Besides regulating haematopoiesis, GM-CSF is a potent modulator of innate immunity, promoting the proliferation, differentiation, activation and/or survival of macrophages and neutrophils (271, 272). Thus, it has been suggested that any potential benefits of GM-CSF based therapies in COVID-19 patients are likely to arise from the suppression of hyperactive innate immune responses (270, 273). In line with this idea, we suggest that, by suppressing emergency granulopoiesis, neutralisation of GM-CSF would dampen the systemic inflammatory response in COVID-19 patients by reducing the mobilisation of pro-inflammatory immature neutrophils into the circulation. Moreover, as immature neutrophils exhibit impaired anti-microbial responses (274, 275), an additional benefit of a more mature circulating neutrophil pool could be a reduced susceptibility to nosocomial infections.

## Conclusions

Several studies of circulating and pulmonary immune cells in patients with COVID-19 have suggested that a hyperactive and dysregulated neutrophil response underpins the development of severe disease. The neutrophil response, including excessive NET generation with compromised removal of cfDNA, and higher frequencies of immature neutrophils with an immunosuppressive phenotype, represent novel therapeutic targets. With few specific treatments approved for COVID-19 other than dexamethasone, we conclude that trials targeting the generation of immature neutrophils, for example by inhibiting the actions of GM-CSF, or improving the removal of cfDNA using gelsolin or AIR DNase™ offer novel and rational approaches to reducing the severity of COVID-19 symptoms. Indeed the advances in our understanding of neutrophil biology and neutrophil mediated pathology as a result of COVID-19 research will likely provide benefit to other clinical conditions considered to be mediated by neutrophils, such as chronic obstructive pulmonary disease.

## Author Contributions 

JH wrote the manuscript and JL critically appraised and revised the manuscript. All authors contributed to the article and approved the submitted version.

## Funding

JH is supported by the National Institute for Health Research (NIHR) Surgical Reconstruction and Microbiology Research Centre (SRMRC). The views expressed are those of the author(s) and not necessarily those of the NIHR or the Department of Health and Social Care.

## Conflict of Interest

The authors declare that the research was conducted in the absence of any commercial or financial relationships that could be construed as a potential conflict of interest.
